# How to Investigate the Origins of Novelty: Insights Gained from Genetic, Behavioral, and Fitness Perspectives

**DOI:** 10.1093/iob/obz018

**Published:** 2019-08-14

**Authors:** C H Martin, J A McGirr, E J Richards, M E St. John

**Affiliations:** 1 Department of Integrative Biology and Museum of Vertebrate Zoology, University of California, Berkeley, CA 94720, USA; 2 Department of Biology, University of North Carolina at Chapel Hill, Chapel Hill, NC 27514, USA

## Abstract

Biologists are drawn to the most extraordinary adaptations in the natural world, often referred to as evolutionary novelties, yet rarely do we understand the microevolutionary context underlying the origins of novel traits, behaviors, or ecological niches. Here we discuss insights gained into the origins of novelty from a research program spanning biological levels of organization from genotype to fitness in Caribbean pupfishes. We focus on a case study of the origins of novel trophic specialists on San Salvador Island, Bahamas and place this radiation in the context of other rapid radiations. We highlight questions that can be addressed about the origins of novelty at different biological levels, such as measuring the isolation of novel phenotypes on the fitness landscape, locating the spatial and temporal origins of adaptive variation contributing to novelty, detecting dysfunctional gene regulation due to adaptive divergence, and connecting behaviors with novel traits. Evolutionary novelties are rare, almost by definition, and we conclude that integrative case studies can provide insights into this rarity relative to the dynamics of adaptation to more common ecological niches and repeated parallel speciation, such as the relative isolation of novel phenotypes on fitness landscapes and the transient availability of ecological, genetic, and behavioral opportunities.

## Synopsis

Como Investigar as Origens da Novidade: Ideias Obtidas a Partir de Perspectivas da Genética, do Comportamento e de Fitness (How to Investigate the Origins of Novelty: Insights Gained from Genetic, Behavioral, and Fitness Perspectives) Biólogos são atraídos pelas adaptações mais extraordinárias do mundo natural, muitas vezes chamdas de novidades evolutivas, mas raramente entendemos o contexto microevolutivo subjacente às origens de novas características, novos comportamentos ou nichos ecológicos. Aqui discutimos ideias obtidas sobre as origens da novidade evolutiva a partir de um programa de pesquisa abrangendo níveis biológicos de organização de genótipo para fitness em pupas do Caribe. Nós nos concentramos em um estudo de caso sobre as origens de novos especialistas tróficos na ilha de São Salvador, Bahamas, e colocamos essa radiação no contexto de outras radiações rápidas. Destacamos questões que podem ser abordadas sobre as origens da novidade evolutiva em diferentes níveis biológicos, como medir o isolamento de novos fenótipos no cenário adaptativo, localizando as origens espaciais e temporais da variação adaptativa que contribuem para a novidade evolutiva, detectando a regulação gênica disfuncional devido à divergência adaptativa, e conectando comportamentos com novas características. As novidades evolutivas são raras, quase por definição, e concluímos que estudos de caso integrativos podem fornecer ideias sobre essa raridade em relação à dinâmica de adaptação a nichos ecológicos mais comuns e especiação paralela repetitiva, como o relativo isolamento de novos fenótipos em cenários adaptativos e a disponibilidade transitória de oportunidades ecológicas, genéticas, e comportamentais. Translated to Portuguese by G. Sobral (gabisobral@gmail.com)

كيفية التحقيق في أصول الحداثة: الأفكار المكتسبة من وجهات النظر الجينية والسلوكية واللياقة البدنية ينجذب علماء الأحياء إلى أكثر التكيفات غير العادية في العالم الطبيعي، والتي يشار إليها غالبًا باسم المستجدات التطورية، ومع ذلك، نادرًا ما نفهم السياق التطوري الجزئي الذي يقوم عليه أصول السمات الجديدة أو السلوكيات أو المنافذ البيئية. هنا نناقش الأفكار المكتسبة في أصول الجدة من برنامج بحثي يشمل المستويات البيولوجية للتنظيم من النمط الوراثي إلى اللياقة البدنية في أسماك pupfish. نحن نركز على دراسة حالة لأصول المتخصصين في التغذية في جزيرة سان سلفادور، جزر البهاما، ونضع هذا التنوع في سياق زيادة التنوع السريعة الأخرى. نسلط الضوء على الأسئلة التي يمكن مخاطبتها حول أصول الحداثة على مستويات بيولوجية مختلفة، مثل قياس عزلة الظواهر الجديدة على مشهد اللياقة البدنية، وتحديد موقع الأصول المكانية والزمانية للتنوع التكيفي الذي يسهم في الحداثة، واكتشاف تنظيم الجينات المختلة وظيفيًا بسبب القدرة على الاختلاف التكيفي، وربط السلوكيات مع السمات الحديثة. المستجدات التطورية نادرة، بحكم تعريفها غالبا، ونستنتج أن دراسات الحالة التكاملية يمكن أن تقدم نظرة ثاقبة في هذه الندرة بالنسبة لديناميات التكيف مع المجالات البيئية الأكثر شيوعًا والإنتواع المتوازي المتكرر، مثل العزلة النسبية للأنماط المظهرية الجديدة على مناظر اللياقة البدنية والتوافر العابر للفرص الإيكولوجية والجينية والسلوكية. translation by A.A. Elbassiouny ( ahmed.elbassiouny@utoronto.ca )

## Introduction

One of the most fundamental biodiversity patterns across the tree of life is the highly uneven distribution of evolutionary novelty (Alfaro et al. 2009; Rabosky and Alfaro 2010; Wagner et al. 2012; Wellborn and Langerhans 2015), i.e., new structures or modifications of existing structures taking on new adaptive functions or new ecological roles (Mayr 1960; Muller and Wagner 1991; Moczek 2008). This pattern is well-known at the macroevolutionary level and is traditionally understood as a direct result of ecological opportunities during adaptive radiation in the form of new environments, key innovations, or mass extinctions (Simpson 1944; Schluter 2000; Losos 2009; Stroud and Losos 2016). However, experimental evolution studies demonstrate that the evolution of novelty is often contingent on pre-existing genetic variation that may steer populations down alternate evolutionary trajectories (Thornton et al. 2003; Blount et al. 2008, 2012; Lozovsky et al. 2009). Macroevolutionary patterns also suggest that ecological opportunity is only weakly associated with the evolution of novel niche specialists (Givnish et al. 1997; Roderick and Gillespie 1998; Erwin 2015; Harmon and Harrison 2015). For example, adaptive radiations sometimes occur long before the existence of ecological opportunity (Schuettpelz and Pryer 2009; Wilson et al. 2012) and often do not exhibit an early burst of trait diversification as predicted by the ecological opportunity hypothesis (Harmon et al. 2010; Landis and Schraiber 2017). Thus, there is a major gap in our understanding of how eco-evolutionary dynamics and microevolutionary processes in nature translate into macroevolutionary patterns of novelty during adaptive radiation (Arnold et al. 2001; Erwin 2015; Higham et al. 2016; Martin and Richards 2019).

Evolutionary novelty is difficult to define and indeed some authors conclude only that “you know it when you see it” (Moczek 2008). Here we broadly define novelty as a new structure (resulting from modification of an existing gene regulatory network) or modification of an existing structure for a new function or ecological role (Moczek 2008; Shubin et al. 2009; Martin and Wainwright 2013a). However, novelty is recognized even more broadly across all levels of biological organization, from *de novo* genes (Ding et al. 2012) to gene expression patterns (Pasquier et al. 2017), morphological traits (Prud’homme et al. 2011; Allf et al. 2016; Hernandez et al. 2017; Davis et al. 2019), behaviors (Sol and Lefebvre 2000; Arnegard and Carlson 2005), and ecological niches (Mckaye and Kocher 1983; Givnish et al. 1997; Burress et al. 2013; Martin and Wainwright 2013a). Novelties can also be deconstructed into their underlying biological processes, encompassing not just environmental factors such as ecological opportunity (Wellborn and Langerhans 2015; Stroud and Losos 2016), but the spatiotemporal origins and sources of adaptive genetic variation contributing to novelties (Möst et al. 2018; Nelson and Cresko 2018), gene expression networks contributing to novel traits (Monteiro and Podlaha 2009), behaviors associated with novel ecological niches (Janovetz 2005; Whitford et al. 2019), and the fitness landscape selecting for novel phenotypes (Arnold et al. 2001). Here we apply this deconstruction approach to our case study of the origins of novelty during adaptive radiation in San Salvador pupfishes and discuss how investigation of these various levels of biological organization can provide insights into the origins of novelty. We also place our case study in the context of other rapid radiations for these various biological processes. We specifically distinguish insights about novel traits, behaviors, and ecological niches as a separate line of inquiry from the large literature investigating more common examples of adaptive traits, behavioral ecology, repeated parallel speciation, and niche diversity, while acknowledging that the definition of evolutionary novelty is often blurry and may be defined, in part, by rarity (Moczek 2008; Wagner and Lynch 2010; Hallgrimsson et al. 2012).

## Why study rare radiations?

If we imagine the ideal natural experiment for studying the evolution of novelty, we would want many replicated, identical environments all colonized by our lineage of interest in which our focal novel trait, behavior, or niche has only evolved in some places. This would provide a large sample of experimental and control environments and populations to test the historical conditions and evolutionary trajectories that give rise to novelty. Indeed, this setup is startlingly close to Lenski’s long-term evolution experiment (LTEE) which provides a landmark study of the evolution of novel citrate-feeding in *Escherichiacoli* despite identical starting conditions across 12 replicate laboratory cultures (Lenski and Travisano 1994; Blount et al. 2008; Meyer et al. 2012). This setup also comes close to describing natural systems that contain what we call “microendemic” radiations of novel ecological specialists. Microendemic radiations occur when a widely distributed species, frequently an omnivore or generalist, has radiated in sympatry in only one or a few locations across its range (Richards and Martin 2017). Examples include *Cyprinodon* pupfishes which diversified in sympatry in only two lakes throughout their entire Caribbean and Atlantic range (Humphries and Miller 1981; Holtmeier 2001; Martin and Wainwright 2011), idiosyncratic patterns of sympatric cichlid radiation across isolated crater lakes and among different cichlid lineages (Seehausen 2006; Wagner et al. 2012; Martin et al. 2015; Kautt et al. 2018), multi-trophic level communities of *Pristionchus* nematodes across different species of fig trees (Susoy et al. 2016), highly restricted patterns of endemism in axolotl salamanders in the Mexican highlands (Shaffer and McKnight 1996), novel ecological specialist pygopodid lizards in Australia (Patchell and Shine 1986), and arctic charr radiations containing up to four ecomorphs in only some glacial lakes (Jonsson and Jonsson 2001). In contrast to many classic adaptive radiations which have radiated within a single unique environment (such as the Galapagos or Hawaiian archipelagos [Roderick and Gillespie 1998; Parent and Crespi 2009]) or repeatedly radiated across similar unique environments (such as *Anolis* lizards across the Greater Antilles [Losos 2009] or haplochromine cichlids across East African rift lakes [Kocher 2004]), microendemic radiations are highly spatially restricted despite the apparent suitability and colonization of many similar habitats by closely related outgroups or a single widely distributed species.

Microendemic adaptive radiations of novel specialists are rare because novelty is rare and most isolated environments containing sufficient levels of ecological opportunity for adaptive radiation to occur are large and unique, rather than highly replicated. Most examples of novel ecological specialists are either ancient taxa (Hoving and Robison 2012) or species restricted to unique environments (Reddy et al. 2012), confounding the ability to separate the effects of environment from organismal traits and phylogenetic history. In contrast, microendemic radiations are nested within a larger set of seemingly comparable environments in which radiation and the evolution of novelty did not occur (Martin 2016a), providing the opportunity to investigate how ecological, genetic, and behavioral traits affect the origins of novelty in action in a systematic and relatively controlled manner rather than millions of years after the fact. In addition, useful features in any system for dissecting the evolutionary origins of novelty include a) young taxa with short generation times suitable for crossing in a laboratory environment, b) large and abundant populations in the field suitable for rapid collection of large sample sizes, and c) hardy taxa suitable for large-scale mark-recapture or pedigree studies of fitness in their natural field environment.

## The pattern of trophic novelty and microendemic adaptive radiation in San Salvador pupfishes

The San Salvador pupfish radiation contains two trophic specialist species, a scale-eater (*Cyprinodon desquamator*; Martin and Wainwright 2013) and a molluscivore (*C. brontotheroides*; Martin and Wainwright 2013), and a third generalist species, *C. variegatus*, which feeds on macroinvertebrates and algae (Martin and Wainwright 2011). All three species occur and breed within centimeters of each other in the littoral benthic habitats within some hypersaline lakes on San Salvador Island, Bahamas but remain largely reproductively isolated with low levels of gene flow (within-lake interspecific *F*_st_ = 0.1–0.3; Turner et al. 2008; Martin and Feinstein 2014) and substantial pre-mating isolation (Kodric-Brown and West 2014; West and Kodric-Brown 2015). This clade is nested within all outgroup Caribbean generalist populations, indicating that these specialists evolved from a generalist ancestor on San Salvador Island (Martin and Feinstein 2014; Martin 2016a; Lencer et al. 2017).

Over 50% of the diet of the scale-eating pupfish results from high-speed (10–15 ms) strikes biting scales and protein-rich mucus from other fishes, usually generalist pupfish which make up 95% of the fish community (Martin and Wainwright 2013a; McLean and Lonzarich 2017; St. John and Martin 2019). This specialized niche has evolved more than 19 times in fishes across diverse environments from the deep sea (Nakae and Sasaki 2002) to the Amazon basin (Evans et al. 2017), but to our knowledge scale-eating evolved only once among over 1500 species of atherinomorph and cyprinodontiform fishes (Sazima et al. 1983; Martin and Wainwright 2013a; Kolmann et al. 2018). Thus, the scale-eating pupfish is separated by 168 million years from the most closely related scale-eating specialists (within the East African cichlid radiations), providing a quantitative phylogenetic index of the novelty of this ecological niche relative to other ecological niches (Martin and Wainwright 2013a). Similarly, the molluscivore pupfish displays a unique nasal protrusion consisting of an anterodorsal extension of the head of the maxilla and nasal bones which may function to leverage *Cerithium* snails loose from their hard shells (Hernandez et al. 2017; St. John et al. in preparation). This nasal protrusion and ecological niche are also unique among cyprinodontiforms which rarely specialize on hard-shelled prey (Martin and Wainwright 2013a).

The San Salvador radiation exhibits oral jaw diversification rates over 1400 times faster than generalist pupfish populations on neighboring Bahamian islands (Martin 2016a), similar to classic adaptive radiations, yet it is less than 15,000 years old (Turner et al. 2008; Martin and Wainwright 2011), the most recent drying of San Salvador’s hypersaline lakes during the last glacial maximum (Hagey and Mylroie 1995). Generalist pupfish populations on seven neighboring islands showed no increased variance in craniofacial skeletal traits or dietary stable isotopes of carbon and nitrogen, despite colonizing nearly identical hypersaline lake habitats lacking predatory and competitive fish species (except for *Gambusia hubbsi* and *Atherinomorus stipes* as on San Salvador [Martin 2016a]). Lake areas, depths, carbonate geology, and genetic diversity of generalist populations in these neighboring hypersaline lakes were also comparable to San Salvador. The only ecological difference was an increase in macroalgal species richness in a few lakes containing trophic specialists on San Salvador; however, these few additional species comprised <1% of the total macroalgal biomass (Martin 2016a). Thus, despite exceptional trait diversification rates and ecological novelty, any differences in ecological opportunity on San Salvador relative to neighboring islands without microendemic radiations appear to be either subtle or nonexistent. Examination of a microendemic radiation at this microevolutionary scale provides a surprising counterexample to the assumptions of the ecological theory of adaptive radiation (Schluter 2000; Losos 2010).

There is a second sympatric radiation of *Cyprinodon* trophic specialists endemic to Laguna Chichancanab in the central Yucatán, Mexico. Similarly, this saline lake contains at least five endemic pupfish species but a different set of trophic specialists, including a large piscivore *C. maya* and a small open-water zooplanktivore *C. simus* (Humphries and Miller 1981; Horstkotte and Strecker 2005; Strecker 2006a), and exhibits exceptional diversification rates over 100 times faster than allopatric generalist species in a different set of traits (Martin and Wainwright 2011). Similar to the San Salvador Island lake system, Laguna Chichancanab is 20 km long with only a single macroalgae species (*Chara* spp.) and one competing fish species, *Gambusia sexradiata* (Humphries and Miller 1981). Unfortunately, this radiation has collapsed due to invasive oreochromine cichlids and Mexican tetra (*Astyanax* sp.) and all trophic specialists may now be extinct in the wild (Schmitter-Soto 1999; Strecker 2006b; Martin 2016a).

**Fig. 1:**
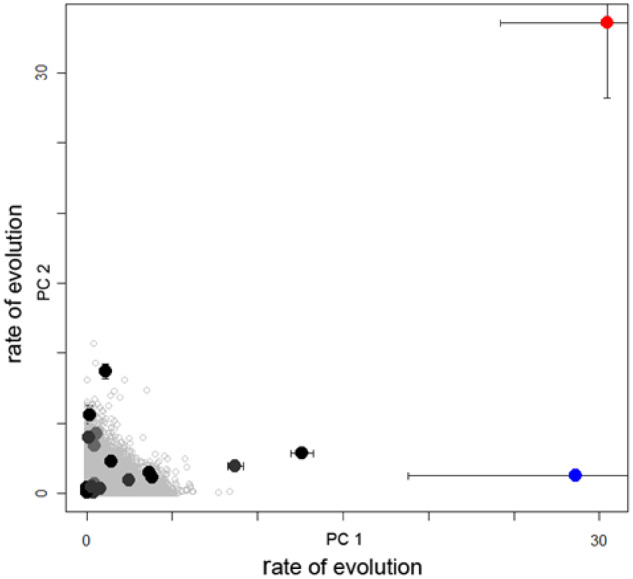
The San Salvador (blue dot) and Laguna Chichancanab (red dot) sympatric radiations of trophic specialists are outliers in trait diversification rates among all Cyprinodontidae clades. Relative rates of trait diversification across all sampled clades of *Cyprinodon* pupfishes and closely related outgroups on the first and second principal component axes for 16 functional traits are shown. All clades with allopatric distributions are shown in black; neutral rates of trait diversification simulated under a Brownian motion model on the Cyprinodontidae tree are shown as open gray circles. Error bars indicate 95% confidence intervals for rate estimates estimated from a random sample of trees from the posterior distribution of the ultrametric phylogeny. Modified from Martin and Wainwright (2011).

In conclusion, ecological opportunity in the form of isolated lakes with few competing fish species is clearly necessary for adaptive radiation but does not appear to fully explain the microendemic distribution of novel ecological specialists among Caribbean pupfishes. Despite many replicated hypersaline lakes of comparable size and ecology colonized by generalist pupfish, sympatric radiations of specialists are known from only two locations. These two radiations exhibit exceptional rates of trait diversification outside the ordinary adaptive continuum of Cyprinodontidae pupfishes (Fig. 1) and have adapted to unique trophic niches among all cyprinodontiform and atherinomorph fishes, a pattern characteristic of ecological novelty. This presents an outstanding opportunity to investigate additional factors contributing to the origins of novel trophic specialists during adaptive radiation beyond ecological opportunity.

## How can fitness measurements inform the study of novelty?

The complex mapping between fitness and phenotype or genotype, known as the adaptive landscape, provides a foundational bridge connecting micro- and macroevolution (Arnold et al. 2001). Indeed, we conceptualize novelty as accessing novel fitness peaks or new adaptive zones, collections of similar ecological niches in new regions of phenotypic space (Simpson 1944; Hallgrimsson et al. 2012). Theoretical simulations and models for the evolution of new species are frequently based on the idea of divergent or disruptive selection driving phenotypic divergence; however, there are few models for the origins of ecologically novel species (Gavrilets and Losos 2009; Gavrilets 2014; Kagawa and Takimoto 2017). Measurements of the adaptive landscape can be used to predict when and where novelty will occur; however, direct measurements of this surface are rare—particularly for intermediate phenotypes of more than two species (Schluter 1994; Schemske and Bradshaw 1999; McBride and Singer 2010; Martin and Wainwright 2013c; Arnegard et al. 2014; Keagy et al. 2016).

We can gain insights into the fitness landscape for novelty by returning to a landmark study on the LTEE (Korona et al. 1994; Blount et al. 2008; Meyer et al. 2012). Each clonal population of *E. coli* initially climbed one or more similar fitness peaks for adapting to a uniform suspension environment in the laboratory. However, a duplication event enabled one strain to cross a fitness valley and access a new higher fitness peak for citrate metabolism (Blount et al. 2012). Thus, even within such a simple environment, different resources corresponding to different fitness peaks exhibit varying degrees of accessibility and isolation relative to different genetic backgrounds descended from the initial founding population of *E. coli*. Similar laboratory experiments have also demonstrated the repeatability of adaptation to various ecological niches (Rainey and Travisano 1998; Jasmin and Kassen 2007; Melnyk and Kassen 2011) whereas field experiments rarely focus on more than two closely related populations occupying similar ecological niches such as different habitats or different color morphs (Schluter et al. 2003; Bolnick 2004; Barrett et al. 2008, 2019).

### The pattern in San Salvador pupfishes

**Fig. 2:**
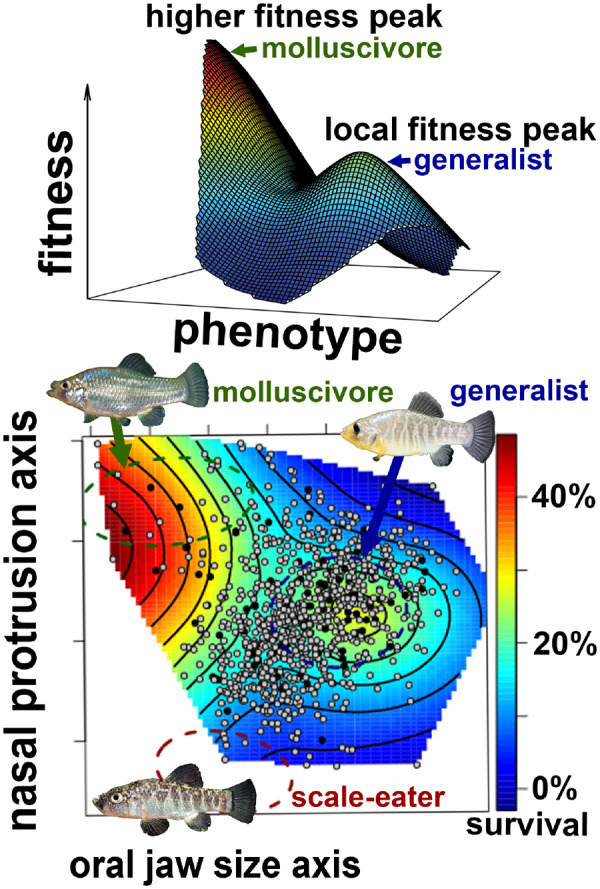
Empirical fitness landscape for San Salvador pupfishes. Heat map in 3-D and 2-D shows probability of F2 hybrid survival in a high-density field enclosure for 3 months. The surface is estimated using generalized cross-validation of a thin-plate spline fit to the raw survival data using the Fields package (Fields Development Team 2006) in R (R Development Core Team 2011). Each F2 hybrid (survivor: black; death: gray)) is plotted within a linear discriminant morphospace for 16 traits separating the lab-reared phenotypes of the three parental species, indicated by 95% confidence ellipses and photographs/arrow, on two discriminant axes with dominant loadings of lower jaw length on the *x*-axis and nasal protrusion distance and angle on the *y*-axis. Modified from Martin and Wainwright (2013c).

We use empirical field measurements of the fitness landscape to study the evolution of novelty in San Salvador pupfishes (Fig. 2). Relative to the gold standard of experimental evolution studies measuring population mean fitness by directly competing derived and ancestral populations (Blount et al. 2012; Wiser and Lenski 2015), our estimates of fitness in pupfishes are substantially restricted, covering only survival and growth rates over a partial generation and not directly measuring lifetime reproductive success. However, even with limited fitness proxies, these landscapes appear to be complex and vary substantially among different phenotypes corresponding to the generalist and specialist phenotypes in each lake (Martin and Wainwright 2013c; Martin 2016b). Our key advantage here is to test the fitness of not only the parental phenotypes, but also the hybrids of each parental type to sample intermediate phenotypes rarely found in natural populations and across a much wider morphospace due to transgressive hybrid phenotypes, following the approach of classic early studies (e.g., Schemske and Bradshaw 1999; Schluter 1994). We so far have avoided comparing hybrid fitness to parental fitness to avoid any unforeseen consequences of hybrid genetic incompatibilities (see later sections).

The magnitude of stabilizing and disruptive selection gradients within the fitness landscape driving adaptive radiation in San Salvador pupfishes (−0.15 to 0.43; Table 1 in Martin and Wainwright 2013c) falls within the range of standardized nonlinear selection gradients estimated in other systems (all taxa: −2.2 to 2.5; vertebrates only: −0.8 to 0.7; Kingsolver et al. 2001, following Fig. 5 in Martin 2012). This suggests that the local strength of disruptive or stabilizing selection on novel trophic specialist phenotypes does not stand out from studies of local adaptation or habitat divergence in other systems (Kingsolver et al. 2001). However, three key results about the broader topography of the complex fitness landscape for novel trophic specialists have emerged from these experiments. First, hybrids resembling the generalist appear to be isolated on a fitness peak for both survival and growth rates (Fig. 2; Martin and Wainwright 2013c). This indicates that strong stabilizing selection is limiting phenotypic diversification of generalist populations in hypersaline lakes on San Salvador Island. Stabilizing selection may also be widespread across Caribbean generalist populations given the similar hypersaline lakes across neighboring islands, providing an explanation for the rare evolution of ecological novelty in this system due to this classic problem of isolation on a local fitness optimum (Gavrilets 2004; Weinreich and Chao 2005). Second, hybrids resembling the molluscivore occupy a higher fitness peak for survival, separated by a small fitness valley from the generalist peak (Fig. 2). This explains the rapid trait diversification rates observed in this system: if a population escapes an isolated fitness peak, trait divergence can occur rapidly on a neighboring peak which may be even higher. Again, this is a classic problem in navigating complex fitness landscapes: a local optimum may not be the highest optima in the surrounding region (Kauffman and Levin 1987; Burch and Chao 1999). Third, hybrids resembling the scale-eaters showed the lowest fitness for both survival and growth rates across different densities and frequencies of hybrid phenotypes, two different lake environments, and two different time periods and seasons (Martin 2016b; Martin and Wainwright 2013c; Martin and Gould in preparation). All evidence gathered so far from two independent field experiments indicates that scale-eater phenotypes are isolated by a large fitness valley from generalist and molluscivore phenotypes and are not connected by fitness ridges in any trait dimension examined, as hypothesized by Gavrilets (1997, 1999). This suggests that the rare evolution of the scale-eating trophic niche within cyprinodontiforms is reflected in the isolation of this fitness peak across a large, multivariate fitness valley. Large fitness valleys isolating the novel feeding niche of scale-eating are also consistent with biomechanical constraints given the high performance demands and low caloric benefits of repeatedly performing high-speed scale-biting strikes on evasive fish prey for only a mouthful of scales and mucus (Sazima 1983; Janovetz 2005; Kolmann et al. 2018).

### Insights gained about novelty

Field measurements of fitness provide an estimate of direct and indirect selection gradients for a suite of traits given a particular environment, performance, or manipulation (Lande and Arnold 1983). These estimates can be used to reconstruct a fitness landscape for a particular environment and project phenotypes observed in nature onto this complex nonlinear space. Thus, fitness measurements of novel phenotypes can provide insights into 1) the selective environment driving trait divergence and may help to 2) quantify the relative isolation of novel phenotypes on the fitness landscape. This can traditionally help to pinpoint the agents of selection and major axes of selection on traits within a radiation (Brodie 1995; Benkman et al. 2001; Calsbeek and Irschick 2007; Svensson and Calsbeek 2012). However, measuring the broader topography of fitness landscapes, particularly using phenotypic manipulations, is a powerful yet highly challenging approach to understanding the relative isolation and rarity of different fitness peaks corresponding to novel phenotypes.

## How can investigating the genetic basis of traits inform the evolution of novelty?

Novel traits are often either temporally or spatially rare, but is the genetic variation underlying such traits also rare? One approach to studying the evolution of novelty is to identify which loci are associated with a novel trait of interest and reconstruct the spatial and temporal origins of the genetic diversity within those regions. One might intuitively think that novel traits are constrained in their evolution across time and space because of the need for *de novo* mutations, which will be 1) spatially rare because they arise in a single population/location and 2) temporally rare because the waiting times for relevant *de novo* mutations are expected to be long (Kimura 1983; Hermisson and Pennings 2005; Barrett and Schluter 2008). However, if novel traits depended solely on *de novo* mutations it would be difficult to explain the rapid evolution of novel ecological specialist species. Microendemic radiations are particularly tractable for investigating the genetic basis of novel adaptive traits because they are nested within a larger set of outgroup populations with ongoing gene flow, which enables divergent genomic regions to be located with high precision, frequently centering on only a single gene (McGirr and Martin 2016; Richards et al. 2018). They are also generally young enough for controlled crosses between species enabling quantitative genetic mapping (Shaw and Lesnick 2009; Martin et al. 2017).

Standing genetic variation present across a group’s range or introduced through hybridization with a divergent lineage via introgression might provide a better source of variation for the rapid evolution of novel traits. Older standing variation that has already been filtered and shaped by selection in its native genetic and ecological backgrounds might allow for the evolution of novel traits more quickly than *de novo* mutations. Variation derived from recent hybridization might also be more potent than standing genetic variation in facilitating rapid evolution of novelty because hybridization between several distinct lineages may result in new combinations of alleles from the parental genomes that lead to transgressive phenotypes in the offspring (Seehausen 2004; Marques et al. 2019). Evidence is mounting in many classic examples of adaptive radiation, including African cichlids (Genner and Turner 2012; Meier et al. 2017; Meyer et al. 2017; Irissari et al. 2018; Poelstra et al. 2018), Darwin’s finches (Lamichhaney et al. 2015, 2016), *Heliconius* butterflies (Heliconius Genome Consortium 2012; Enciso-Romero et al. 2017), and stickleback (Colosimo et al. 2005; Nelson and Cresko 2018) that alleles contributing to ecological divergence and/or reproductive isolation are often older than the actual divergence events. Thus, sympatric radiations of ecological specialists may frequently adapt to their new niches using standing genetic variation, which may be left over from previous cycles of radiation and species collapse (Turner 2002; Taylor et al. 2006; Martin et al. 2016; Nelson and Cresko 2018).

### The pattern in San Salvador pupfishes

The novel traits of the scale-eating and molluscivore pupfishes are endemic to San Salvador Island, but intriguingly we find evidence that nearly all genetic variation underlying these traits exists in other parts of the Caribbean. At the genome-wide level, we see extensive evidence of gene flow among Caribbean lineages of pupfish (Martin 2016a); 3.1% and 3.7% of the scale-eater and molluscivore genomes, respectively, appear to be derived from introgression (Richards et al. 2018). This level of introgression is similar to cichlid radiations in which hybridization has also been investigated as a source of genetic variation underlying diversification (e.g., 1–4%; reviewed in Richards et al. [2019]; also see Malinsky et al. 2015; Kautt et al. 2016; Meier et al. 2017; Richards et al. 2018; Poelstra et al. 2018). Furthermore, some of these introgressed regions may have played an important role in this radiation as they are strongly diverged between the specialists, show signatures of a hard selective sweep, and are centered on genes with known craniofacial effects in model organisms (Richards et al. 2018). For example, we found a signature of adaptive introgression from *C. laciniatus*, a generalist population 200 km away on New Providence Island, into the molluscivore in a regulatory region of the gene *ski*. *Ski* is a proto-oncogene that causes depressed nasal bridges and shorter, thicker mandibles in knockout mice (Berk et al. 1997; Colmenares et al. 2002), traits that phenocopy the nasal protrusion and shorter and thicker dentary bone in molluscivore pupfish (Lencer et al. 2016; Hernandez et al. 2017). We found similar signatures of adaptive introgression of other craniofacial genes into the scale-eater from the same *C. laciniatus* population (Richards et al. 2018). Thus, ancient reservoirs of standing genetic variation in distant generalist populations contributed different sets of adaptive alleles to the trophic specialists on San Salvador Island (Richards et al. 2018, in preparation).

### Insights gained about novelty

An emerging consensus in the study of rapid radiation and speciation is that adaptive alleles involved in species divergence are often far older than the radiation itself (Nelson and Cresko 2018; Marques et al. 2019). These alleles may also be traced to a particular point in space with sufficient sampling of outgroups and dated to a particular point in time using various levels of sophistication, from simply counting point mutations within the region to joint inference of selection and timing of hard selective sweeps (Laurent et al. 2015; Oziolor et al. 2019). Thus, investigating the genetic basis of novel adaptive traits can provide strong inferences regarding 1) selection on the trait of interest, and 2) evolutionary history of the trait in space and time. For example, existing radiations may be remnants of past radiations (Joyce et al. 2005) or repeated parallel speciation may result from repeated selection on standing genetic variation (Colosimo et al. 2005; Schluter and Conte 2009; Conte et al. 2015). Using only genetic data, these classic case studies inform our understanding of the environmental context of novelty.

## How can gene expression inform studies of novel traits and behaviors?

Changes in gene expression are an important source of phenotypic variation during the evolution of novel traits (Carroll 2008). RNAseq is now routinely used to identify expression patterns that are unique to populations with novel traits (Monteiro and Podlaha 2009; Renaut et al. 2009; Manousaki et al. 2013; McGirr and Martin 2016). The most common approach is to sample from developing tissues that give rise to a particular trait of interest and search for genes that show unique expression patterns in the focal species relative to closely related species or populations without the trait.

**Fig. 3:**
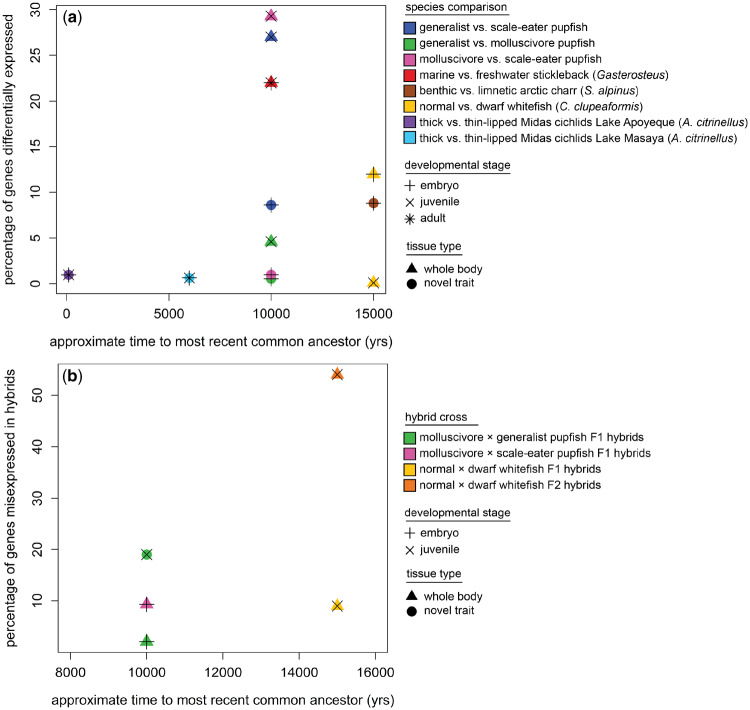
Comparison of **a**) differential gene expression and **b**) hybrid gene misexpression among recent radiations and San Salvador pupfishes. a) The percentage of differentially expressed gene transcripts ranges from 1% to 30% among recent radiations, but the San Salvador trophic specialists with novel traits (blue, green, and pink triangles) do not stand out. b) Few studies have examined hybrid gene misexpression in non-model systems; however, molluscivore×generalist F1 hybrids exhibit substantial gene misexpression in their craniofacial tissues, including their unique nasal protrusion (green circle), relative to lake whitefish F1 hybrids (yellow triangle; but not F2 whitefish hybrids (orange triangle) which may be expected to exhibit transgressive expression patterns). Data from McGirr and Martin (2017, 2019); Jones et al. (2012); Ahi et al. (2014); Renaut et al. (2009); and Manousaki et al. (2013).

However, the number of genes differentially expressed between species with similar divergence times varies widely across gene expression studies in fishes (Fig. 3a). The different methods used to quantify gene expression across studies explain some of this range, but even studies using similar methods show significant variation. For example, 22% of genes in adult tissues were significantly differentially expressed between marine and freshwater stickleback populations (Jones et al. 2012). In lake whitefish, 11% of genes in whole-body juvenile tissues were differentially expressed between species (Renaut et al. 2009). In San Salvador pupfishes, between 4% and 29% of genes were differentially expressed in generalists versus molluscivores and molluscivores versus scale-eaters, respectively, in whole larvae sampled 8 days post-fertilization (8 dpf; McGirr and Martin 2017).

Clearly, in nearly all cases, other methods need to be implemented alongside differential expression comparisons to identify individual genes influencing new traits. A standard approach now is to cross-reference lists of differentially expressed genes with differentiated genomic regions showing both relative and absolute divergence between species or populations (Nachman and Payseur 2012; Cruickshank and Hahn 2014), followed by description of statistically over-represented (enriched) gene ontology categories within the final list of genes. Ultimately, the elusive goal of locating causative genetic variation will require a suite of complementary genomic approaches (such as genome-wide selection scans, differential expression between species, allele-specific expression in F1 hybrids, and genome-wide association mapping) and functional experiments manipulating expression or candidate genetic variants *in vivo* (Hoekstra et al. 2006; Miller et al. 2007; Linnen et al. 2009; Chan et al. 2010). It is important to also recognize that most novel traits are probably controlled by many small-effect loci. This makes identifying causal alleles contributing to novel polygenic traits (quantitative trait nucleotides: QTNs) virtually impossible. Instead, studying higher levels of biological organization such as gene expression networks or simply focusing on the quantitative genetics of novel phenotypes may be more productive research directions (Rockman 2012); however, given the initial successes of the “QTN” program in finding moderate- and large-effect loci, it is very difficult to abandon.

Nonetheless, gene expression studies also offer a tool to detect potential genetic incompatibilities due to the adaptive divergence of novel traits. If closely related species can be crossed to generate F1 hybrids, then RNAseq of developing tissues relevant to the novel trait of interest can be used to identify genes that are misregulated in hybrids relative to both parental populations. Identifying genes that are differentially expressed in hybrids relative to both sets of parents can point to potential (Bateson)–Dobzhanksy–Muller incompatibilities (DMIs) within the genetic regulatory networks shaping novel traits (Renaut and Bernatchez 2011; Mack and Nachman 2017). For example, the same study of lake whitefish mentioned above found that 9% of genes were misregulated in whole-body juvenile tissues resulting from interspecific F1 hybrids and 54% of genes were misexpressed in F2 hybrids (Renaut et al. 2009). Hybrid gene misexpression has been linked to higher levels of male sterility in adult mice (Mack et al. 2016) and may be an underappreciated feature of compensatory evolution and divergent selection in different populations, contributing to growing evidence of widespread genetic incompatibilities even between recently diverged species (Ortiz-Barrientos et al. 2007; Renaut and Bernatchez 2011; Schumer et al. 2014; Mack et al. 2016).

### The pattern in San Salvador pupfishes

We found high levels of F1 hybrid gene misexpression in the craniofacial tissues of San Salvador pupfishes from late-stage larvae (17 dpf), reaching approximately 19% of genes (McGirr and Martin 2019). This was much higher misexpression than F1 lake whitefish hybrids which showed 9% of genes misexpressed, but lower than F2 whitefish hybrids which showed 54% of genes misexpressed (Renaut et al. 2009). In a second independent study, this high level of misexpression within the San Salvador radiation between trophic specialists equals or exceeds misexpression in crosses between more highly diverged generalist populations across the Caribbean (McGirr and Martin 2019). Many of these genes show evidence of hard selective sweeps in regulatory regions and were involved in skeletal development, brain development, and metabolism, key axes of ecological divergence within the San Salvador radiation (McGirr and Martin 2019). Genes may be misregulated in F1 hybrids as a result of compensatory evolution of *cis*- and *trans*-acting elements in different species of pupfish, even those coexisting within the same environment. Indeed, this has broad implications for ecological speciation in general if divergent selection on adaptive traits may also result in reproductive isolation due to gene misexpression in hybrids. Interestingly, the high level of hybrid misexpression observed between our novel trophic specialists appears to stand out relative to other speciation systems of similar age (e.g., Renaut et al. 2009), suggesting that highly novel phenotypes may be more likely to lead to regulatory incompatibilities. However, more studies are needed to understand how hybrid gene misexpression relates to the evolution of novel phenotypes.

### Insights gained about novelty

Gene regulation is one of the fundamental ways that evolution shapes existing structures for new functions. However, studies of differential expression between species or novel tissue types rarely result in a tractable list of candidate genes. Furthermore, existing studies of rapid radiations and speciation show a large range of differential gene expression and no clear associations with novelty (Fig. 3a). Conversely, the few studies of gene misexpression in hybrids suggest that novel traits may stand out in displaying higher amounts of misexpression among recently diverged sympatric species (Fig. 3b). Studies of hybrid misexpression can highlight potential genetic incompatibilities resulting from ecological or sexual divergence between species, even within rapidly diverging sympatric radiations. Thus, 1) gene expression studies can help refine the list of candidate regions underlying the novel trait of interest but must be combined with genome scans, genome-wide association studies, and functional studies. 2) Gene misexpression studies in hybrids can identify potential genetic incompatibilities due to the evolution of novel traits. These studies may highlight genetic networks that lead to lower hybrid fitness as a result of selection for novel traits, but require the ability to cross species and generate F1 hybrids. Nonetheless, quantifying gene expression and misexpression in hybrids may be one of the most tractable ways to link novel traits to reproductive isolating barriers at the genetic level in the form of DMIs.

## How can behavioral studies inform the origins of novel ecological niches?

Behavior plays a fundamental role in adaptation to new ecological niches, the origins of evolutionary innovations, and novel traits. There is still debate about the relative roles of behavior and morphology in driving the origins of novel ecological niches and ecological speciation, i.e., behavior-first or morphology-first adaptation to novel resources (Sol and Lefebvre 2000; Duckworth 2009; Zuk et al. 2014). This has been tested at macroevolutionary scales and through microevolutionary comparisons among populations (Todd Streelman et al. 2003; Losos et al. 2004; Munoz and Losos 2018). Here we discuss insights gained in testing the behavior-first hypothesis for the novel behavior and ecological niche of scale-eating within the San Salvador radiation.

The origins of scale-eating are still unknown; however, there is no shortage of hypotheses, all of which involve behavior-first explanations. The three main hypotheses are 1) the algae-grazer hypothesis, 2) the cleaner hypothesis, and 3) the aggression hypothesis. The algae-grazer hypothesis proposes that scale-eating arose due to incidental ingestion of scales while foraging on algae (Fryer et al. 1955; Greenwood 1965; Sazima 1983). This hypothesis is supported by the fact that many scale-eating species are most closely related to species that primarily forage on algae (Trewavas 1947; Greenwood 1965; Fryer and Iles 1972). While this hypothesis provides a plausible explanation for how scale-eating species began consuming scales, it does not provide a clear explanation for why they began seeking scales from the bodies of other fish. The cleaner hypothesis proposes that the incidental ingestion of scales arose while foraging for ectoparasites on the surface of other fish (Trewavas 1947; Greenwood 1965; Fryer and Iles 1972). Scales have been found in the digestive tracks of some cleaner fish (e.g., Losey 1972; Demartini and Coyer 1981; Sazima 1983). However, there are few examples of primarily scale-eating species that also consume ectoparasites. Finally, the aggression hypothesis suggests that scale-eating arose due to the incidental ingestion of scales during inter- or intra-specific fighting (Sazima 1983). The fact that many scale-eating specialists are characterized as being aggressive provides support for this hypothesis (Peterson and Winemiller 1997; Janovetz 2005). While any of these hypotheses may be plausible, they each provide unique, testable predictions that can be used to decipher the behavior-first origins of scale-eating.

### The pattern in San Salvador pupfishes

The scale-eating pupfish is an excellent species to investigate the potential behavioral origins of scale-eating for two reasons. First, it is the youngest known scale-eating specialist at 10,000 years old (Martin and Wainwright 2011; St. John et al. 2018), providing a window into the microevolutionary origins of scale-eating. Second, previous studies have documented various aspects of pupfish ecology and evolution allowing us to determine which hypotheses were most likely in this system. For example, previous dietary studies and observations of scale-eaters in the wild offer no evidence that scale-eating pupfish consume ectoparasites. Although all San Salvador pupfishes consume macroalgae, the algae-grazer hypothesis does not provide a mechanism for why fish would seek scales from the bodies of other fish. Finally, observations of scale-eating in the wild indicate strong aggression in scale-eaters during foraging and directed at conspecifics—supporting aggression as a possible behavioral origin for scale-eating in this system.

The aggression hypothesis makes a clear and easily testable prediction: scale-eating pupfish should be more aggressive than generalist or molluscivore pupfish. We tested this using behavioral assays to measure aggressive behaviors (e.g., number of attacks performed) toward a mirror image for generalist, scale-eater, and molluscivore pupfishes. This simple and widely used assay may not always elicit the same response as a live opponent (Li et al. 2018a, 2018b), thus we also measured aggression toward conspecific and heterospecific fish in live paired trials. We unexpectedly found increased levels of aggression in both scale- and snail-eating pupfish across multiple contexts. This is particularly striking given that scale-eating involves high-speed ramming strikes on pupfish prey, an escalation of typical aggressive behavior, whereas consuming snails does not require high-speed strikes nor conspecific aggression, only increased stability of oral and pharyngeal jaws (Hernandez et al. 2017; St. John and Martin 2019). These results do not support the aggression hypothesis and indicate that both specialists may exhibit high levels of aggression due to trophic specialization or due to the indirect effects of selection on other traits (St. John et al. 2018).

Gene expression studies can also lend context to the study of behavior-first hypotheses. For example, we found that only seven genes were differentially expressed in aggression-related ontologies between specialist pupfish and generalist pupfish and only two genes (*rnf14* and *crebrf*) were differentially expressed in both specialists compared with generalists. However, all seven genes have pleiotropic effects on other divergent traits in this system, including craniofacial morphology, protein degradation pathways, and melanin pigmentation (St. John et al. 2018). Thus, consideration of differentially expressed genes provides many possible avenues for indirect selection for increased aggression due to divergent ecological selection on morphological or physiological traits or sexual selection for divergent mating coloration through reproductive character displacement (Pfennig and Pfennig 2012).

### Insights gained about novelty

Behavior is central to any investigation of novelty; however, it often remains unclear whether novel behavior is the ultimate driver of novel resource use or whether the evolution of a new morphological structure or physiological process enables access to new resources through existing behaviors. Studies of behavioral ecology in the context of novelty can provide insights into this longstanding question by 1) helping to reconstruct the origins of behaviors associated with novelty using phylogenetic comparative methods. For example, novel behaviors, such as increased aggression during scale-eating, are present in a non-sister trophic specialist species (i.e., the molluscivore pupfish), thus shifting the role of aggression in scale-eating to an association with trophic specialization in general. Conversely, 2) consideration of pleiotropy in candidate genes for observed behavioral differences can suggest whether behavioral phenotypes may be an indirect effect of selection on other adaptive phenotypes. Testing the causal effects of behavior on the origins of novelty remains a formidable challenge, but behavioral ecology studies coupled with an understanding of the pleiotropic effects of gene function on behavior and other phenotypes are necessary components of these investigations.

## Future directions and predictions for the study of evolutionary novelty

In this review, we examined processes underlying the origins of novelty at the level of fitness, genetic variation, gene expression, and behavior. These perspectives and emerging results suggest distinct predictions for investigations of novelty in other systems. Here we speculate about general patterns underlying the origins of evolutionary novelties based on the unusual features and biological processes observed in our case study of the origins of novel trophic specialists in San Salvador pupfishes.

First, we predict that novel traits, behaviors, and ecological niches may be more isolated on the fitness landscape, i.e., these phenotypes may be separated by wider and deeper fitness valleys from other phenotypes occupying more common ecological niches. Further investigation of the relative isolation of performance optima through biomechanical and kinematic models (Holzman et al. 2012; Tseng 2013; Stayton 2019; St. John and Martin 2019) and fitness optima, particularly in natural field environments (Keagy et al. 2016; Pfaender et al. 2016), will help to characterize the rich diversity of organismal performances and ecological niches. We also neglected discussion of how mate preferences may shape phenotypic optima and constrain/accelerate speciation through processes such as sensory drive and magic traits (Martin 2013; Ryan and Cummings 2013; Servedio and Boughman 2017). Ultimately, we think that estimation of the topography of fitness and performance landscapes may predict some of the variation in existing distributions of organismal morphological, ecological, and behavioral diversity. For example, only three performance metrics of turtle shells (strength, righting ability, and hydrodynamics) can largely explain the extant distribution of shell shapes and this joint performance landscape outperforms phylogenetic models (Stayton 2019).

Second, ancient balancing selection is now frequently associated with ecological divergence between populations and repeated parallel speciation (Guerrero and Hahn 2017). However, we predict that novel phenotypes may result from a greater contribution of *de novo* variation or adaptive introgression from specific source populations rather than widespread standing genetic variation. This is expected if larger fitness valleys isolate novel phenotypes, selecting for alleles of larger effect size to cross these valleys which may also exhibit strong negative sign epistasis (Orr 2005; Weinreich et al. 2005). Indeed, there is evidence of larger-effect quantitative trait loci (QTL) for increasing the oral jaw size of the scale-eater, which we interpret as a result of adaptation to a more distant fitness peak across a larger fitness valley than the molluscivore (McGirr and Martin 2016; Martin et al. 2017). The relative contribution of standing genetic variation, introgression, and *de novo* mutation to adaptive phenotypes is an empirical question (Pease et al. 2016) that will vary from one environmental context and ecological niche to another, but may also contain information about the underlying adaptive landscape (Korona et al. 1994; Orr 2005; Carneiro and Hartl 2010). Thus, understanding the relative contributions of standing genetic variation and their effect sizes to an adaptive walk toward a fitness optimum for novel traits can complement investigation of the topography of fitness landscapes and contribute to evidence for the relative isolation of different ecological niches and novel phenotypes.

Third, we predict that adaptive divergence of novel traits may result in more dysfunction in genetic regulatory networks in hybrids, detected as gene misexpression in F1 hybrids, than more typical ecological and morphological divergence observed during local adaptation or repeated parallel speciation. This prediction is related to the snowball effect for genetic incompatibilities with increasing genetic divergence between populations (Matute et al. 2010; Moyle and Nakazato 2010): as the complexity of adaptive divergence between two novel ecological niches increases, the complexity of the underlying genetic regulatory networks should also increase resulting in increased opportunities for genetic conflicts to arise between these diverging populations compared with adaptive divergence between more similar ecological niches, such as habitat divergence or color polymorphism (Linnen et al. 2009; Poelstra et al. 2014). Thus, we predict that more divergent traits or ecological niches should result in more DMIs, greater reproductive isolation due to these DMIs, and more gene misexpression in F1 hybrids.

Fourth, we predict that behaviors often associated with the origins of novel traits and ecological niches may be incidental effects of selection on highly pleiotropic genetic networks. Thus, causality is difficult to establish without fitness, performance, and behavioral ecology studies to understand the direct and indirect targets of selection. The numerous pathways with indirect effects on behavior (and the omnigenic model in general: Boyle et al. 2017) provide important context for considering the scope of potential targets of selection during adaptive divergence.

Clearly, much work remains to establish general patterns about the origins of evolutionary novelties at genomic, gene regulation, behavioral, and fitness landscape scales. The persistence of ecological specialist species across temporal scales is also currently unknown. Although some ecological specialists may be viewed as ephemeral due to stochastically shifting environments (Rosenblum et al. 2012) and incomplete reproductive isolation (Turner 2002; Taylor et al. 2006; Keagy et al. 2016), the relevant adaptive alleles may persist long before and after such cycles of speciation and collapse (Meier et al. 2017; Richards and Martin 2017; Nelson and Cresko 2018; Marques et al. 2019). Moreover, some “classic” adaptive radiations, such as East African cichlids, display similar recent spatiotemporal dynamics and minimal genetic structure underlying extraordinary differences in ecology (Malinsky et al. 2015), thus it is not clear how evolutionary novelty scales with ecological transience or species diversity across the diverse volume of adaptive radiation space (Martin and Richards 2019). However, we think that distinguishing and quantifying the novelty of adaptations and ecological niches within recent microendemic radiations is worthwhile to discover potentially unique biological processes and features of adaptive landscapes which contribute to the extraordinary adaptations and organismal functions that we value as integrative biologists.
